# Supplementary material for: Complexome profiling of the Chlamydomonas psb28 mutant reveals TEF5 as an early PSII assembly factor
Source: Plant Cell. 2025 Mar 17;37(6):koaf055. doi: 10.1093/plcell/koaf055 (PMC12164590; doi:10.1093/plcell/koaf055)
Supplement: koaf055_Supplementary_Data [file koaf055_supplementary_data.zip › psb28_interactive.zip!/plots/intensity/qualityPlots/peptides/PHB1_Cre02.g088000.t1.2.html]

### Cre02.g088000.t1.2

Trivial name: PHB1  
SeqCoverage [median]: 47.90  
Mass: 33.56 kDa  
  
 

| Sequence | MissedCleavage | Mass [Da] | wtIntensity | muIntensity | Score |
| --- | --- | --- | --- | --- | --- |
| AAPNPSQLAGALGAAR | 0 | 1463.78 | 3639.30 | 0.00 | 79.49 |
| ARPSVIQSQSGSK | 1 | 1343.72 | 13191.96 | 14952.07 | 119.52 |
| DLQMVNVGLR | 0 | 1143.61 | 7918.15 | 12538.18 | 150.36 |
| EIAGTISQSANK | 0 | 1217.63 | 13173.63 | 15522.92 | 196.07 |
| EYTAAVEAK | 0 | 980.48 | 3681.25 | 3949.46 | 119.27 |
| GTELNLTEGTSR | 0 | 1276.63 | 0.00 | 4253.02 | 187.64 |
| LIGEAVKQNPAFLTLR | 1 | 1769.02 | 1396.91 | 1858.79 | 59.86 |
| QNPAFLTLR | 0 | 1058.59 | 852.18 | 0.00 | 55.55 |
| QVAQQEAER | 0 | 1057.52 | 4490.16 | 931.24 | 122.13 |
| TLGTDYAER | 0 | 1024.48 | 3638.80 | 5450.34 | 88.95 |
| VLPSIIQETLK | 0 | 1239.74 | 650.71 | 0.00 | 68.73 |
| VLTRPNADKLPEIYR | 2 | 1783.99 | 3440.37 | 6451.34 | 45.01 |
|  |  |  | 56073.4 | 65907.4 |  |
